# Characterization of a fungicidal substance produced by *Eupenicillium brefeldianum* isolated from soil for plant disease control and its significance in nature

**DOI:** 10.1186/1999-3110-55-39

**Published:** 2014-07-22

**Authors:** Yen-Ting Chen, Wen-Hsiung Ko

**Affiliations:** Department of Plant Pathology, National Chung Hsing University, Taichung, Taiwan

**Keywords:** *Alternaria brassicicola*, Competitive saprophytic ability, *Eupenicillium brefeldianum*, Fungicidal substance

## Abstract

**Background:**

A fungus identified as *Eupenicillium brefeldianum* was isolated from soil amended with vegetable tissues.

**Result:**

When grown in liquid medium prepared from the same vegetable tissues, *E. brefeldianum* produced a substance capable of preventing disease development of leaf spots of mustard cabbage caused by *Alternaria brassicicola* and inhibiting the germination of *A. brassicicola* conidia. The inhibitory substance was fungicidal and was very stable under high temperature and extreme pH. It was soluble in polar solvents but not soluble in non-polar solvents, and did not have charges on its molecule. This is the first discovery of the production of a fungicidal substance by this fungus.

**Conclusion:**

Results from this study suggest the possession of a strong competitive saprophytic ability by *E. brefeldianum*, which in turn may explain the widespread occurrence of this fungus in soils.

## Background

Leaf spot of crucifers caused by *Alternaria brassicicola* (Schwein) Wiltshire, *A. brassicae* (Berk.) Sacc., and *A. raphani* Groves & Skolko are of worldwide importance (Farr et al. 1989). These pathogens have caused considerable losses in cruciferous oil production and vegetable crop yield (Humpherson-Jones and Maudo 1982; Kolte et al. 1987). In Taiwan, *A. brassicicola* is the causal organism of leaf spots on crucifers. The disease causes reduction in quality and yield of cruciferous vegetables all year round (Ho et al. 2007).

Control of foliar plant diseases relies heavily on the use of chemical fungicides (Agrios 2005; Hewitt 1998). However, development of alternative methods for the control of these diseases is needed because of the high concerns about the adverse effects of pesticides on human health and the environment (World Health Organization 1990). A project was, therefore, initiated to screen liquid cultures of soil microorganisms directly on leaves of mustard cabbage for ability to control leaf spots caused by *A. brassicicola* (Chen et al. 2011). Liquid culture of a fungus identified as *Eupenicillium brefeldianun* was found to be very effective in controlling the disease in the greenhouse. The fungus is famous for its ability to produce brefeldin A which has multiple functional and biochemical effects on experimental animals (Kamata et al. 1983; Klausner et al. 1992; Nebenfuhr et al. 2002). This fungus is found throughout the world in soil including agricultural, forest, orchard and pasture soils (Keller and Bidochka 1998; Souza-Motta et al. 2003). Little is known about the mechanism of its widespread occurrence in soil. The objectives of the study are to characterize the inhibitory substance produced by *Eupenicillium brefeldianum* and to explain why this fungus is so widespread in nature.

## Methods

### Isolation of soil microorganisms

Soil samples collected from central Taiwan were taken from a depth of 0–10 cm, sifted and moistened to about 65% water-holding capacity (Tsai et al. 2012). Selective media for isolation of fungi, actinomycetes and bacteria were prepared as described previously (Ko et al. 2010b). Approximately 1.3 g soil was mixed with 100 ml sterile distilled water in an Omni mixer chamber at 5000 rpm for 30 s. The suspension was diluted to 10^−4^, 10^−5^ and 10^−6^ for fungi, 10^−5^, 10^−6^ and 10^−7^ for actinomycetes and bacteria to determine the dilution needed to obtain soil suspension essentially free of each group of microorganisms (Ko et al. 2011). A 1-ml aliquot of diluted soil suspension was mixed with 20 ml of a molten selective medium at 45°C in a Petri plate. Five plates were used for each treatment.

Vegetables including fruit of tomato (*Lycopersicon esculentum*), tubers of sweet potato (*Ipomoea batatas*), and leaves and stems of spinach (*Spinacia oleracea*), ong choy (*Ipomoea aquatica*) and common purslane (*Portulaca oleracea*) were purchased from local markets. For soil amendment, about 500 g soil was mixed with 4% each of chopped vegetables in a 1000-ml bottle and incubated at 24°C for at least two weeks before use. To isolate microorganisms with ability to utilize amended nutrients for multiplication, suspension of amended soil was diluted to the concentration pre-determined for each group of microorganisms and plated on the selective medium as described above. After incubation at 24°C for seven days, colonies appeared were individually transferred to 10% V-8 agar (10% V-8 juice, 0.02% CaCO_3_ and 2% agar) plates (Ko et al. 2010a).

### Cultivation of isolated microorganisms in liquid medium

Liquid medium was prepared by grinding 4 g each of the five chopped vegetables in 100 ml water in an Omni mixer at 4000 rpm for 3 min and dispensing 50 ml broth in a 250-ml flask. After autoclaving, each flask was inoculated with two loopfuls of bacterium, or a piece (ca. 4 × 5 × 3 mm) of actinomycete or fungus agar culture. Inoculated flasks were incubated on a shaker for two weeks. After incubation, cultures were separately ground in an Omni mixer at 4000 rpm for 1 min and the mixtures were left on the bench for sedimentation. The fluid portions were used for testing their ability to control the disease.

### Inoculum preparation

*A. brassicicola* isolate Aba-31 was grown on 10% V-8 agar at 24°C under light (Wang et al. 2010) for four to six days for the production of conidia. A conidial suspension was prepared by placing two pieces of culture blocks (ca. 5 × 5 × 3 mm) in 5 ml sterile distilled water in a test tube and by agitating the test tube for 30 s with a Vortex mixer. The concentration of conidia was adjusted to 3 conidial/μl with a Pipetman microliter pipette (West Coast Scientific, Oakland, CA) (Ann et al. 2010).

### Disease control assay of culture fluids

Seeds of mustard cabbage (*Brassica juncea* Coss.) were grown in 8-cm pots containing a mixture of peat moss and vermiculite (9:1, v/v). Two leaves of a four-week-old plant were sprayed to run off with culture fluid of a test microorganism daily three times before inoculation on the fourth day.

Each mustard cabbage leaf was inoculated with five 2-μl drops of conidial suspension of *A. brassicicola* along the edge of the leaf, and a 10-μl drop of molten agar consisting of 1% agar and 1% V-8 juice at 60°C was added to each inoculum drop to fix the inoculum on the target site (Chen et al. 2011). Leaves of mustard cabbage sprayed with liquid medium were inoculated with conidia of *A. brassicicola* and used as a control. Inoculated plants were placed in moist chambers and kept in the greenhouse. The number and the size of lesions that developed at the inoculated sites were recorded three days after inoculation. Two leaves were used for each treatment, and all the experiments were repeated at least twice.

### Extraction of the fungicidal substance

Culture fluid of *E. brefeldianum* isolate V3F-3 was freeze dried. One gram dry powder, obtained from approximately 50 ml of culture fluid was extracted with 25 ml of water, ethanol, methanol, acetone, ethyl acetate, or chloroform in a 250-ml flask by shaking on a shaker for 24 h (Ko et al. 2010b). The mixture was centrifuged at 1500 × g for 5 min to obtain clear extract. For bioassay and characterization of the fungistatic substance, 10 ml extract was evaporated to 2 ml followed by addition of 2 ml water and evaporation to 2 ml again. To test the ability of different solvents to extract the inhibitory substances, 10-fold concentration of the extracts was used.

### Germination tests

To test the effect of the culture extract on spore germination, 10 μl of conidial suspension (2 × 10^4^ spores/ml) of *A. brassicicola* was mixed with 10 μl of extract in a cavity of a sterile eight-cavity slide. Slides with spores were kept moist by placing each on a L-shaped glass rod in a 9-cm Petri plate containing 10 ml sterile distilled water. Germination was recorded after incubation at 24°C for 4 h, and 100 spores were counted in each of the three replicates. All experiments were done twice.

### Characterization of the fungicidal substance

To study the effect of pH on the activity of the fungicidal substance, the pH of the culture extract of *E. brefeldianum* was adjusted from the original 4 to 3 with 1 N HCl, or 5 to 10 with 1 N NaOH (Ko et al. 2010b). To study the stability of the fungicidal substance under extreme pH, the pH of the extract was adjusted to 2 or 12 for 24 h and then readjusted back to 4.

For studying the effect of high temperature on the activity of the fungicidal substance, the extract was treated at 60, 80 or 100°C for 30 min or autoclaved for 15 min. For the study of the effect of the treatment of culture extract with cation exchange resins, anion exchange resins or activated charcoal on its activity against germination of *A. brassicicola*, 5 g of Diaion SK1B cation exchange resins (equivalent to Amberlite 1R-120), Diaion SA 12A anion exchange resins (equivalent to Amberlite 1RA-420; Tai-Young Chemical Co., Kaohsiung, Taiwan) or activated charcoal (Sigma-Aldrich) was washed with 50 ml of distilled water three times by shaking over a six-hour period to remove possible inhibitory substances. Ten milliliter extract was shaken with 1 g cation exchange resins, anion exchange resins or activated charcoal in a 150-ml flask at 100 strokes/min for 24 h and filtered through a Whatman no. 1 filter paper. The filtrate was then used for germination tests (Ko et al. 2010b). In each of the three replicates, 100 spores were counted and all experiments were done at least twice.

## Results

### Effect of culture on leaf spots

Among the 241 microorganisms isolated from soils amended with vegetable tissues tested, liquid cultures from one bacterium and five fungi were able to reduce the disease incidence of leaf spots of mustard cabbage caused by *A. brassicicola* from 90% in the control to 0-30%. Liquid culture of the fungus identified as *E. brefeldianum* was able to completely suppress the disease development and inhibit spore germination of the pathogen (Table 1). Moreover the fungus is famous for its production of brefeldin A (Nebenfuhr et al. 2002) and widespread occurrence in nature (Keller and Bidochka 1998; Souza-Motta et al. 2003). It was, therefore, selected for further study.

**Table 1:** **Effect of liquid culture of** ***Eupenicillium brefeldianum*** **on symptom development on mustard cabbage leaves inoculated with conidia of** ***Alternaria brassicicola*** **and on conidial germination of the pathogen**

	Symptom development^a^		
Treatment	Disease incidence (%)	Lesion size (mm)	Conidial germination^b^(%)
Culture	0	0	0
Water (control)	90	3.7	97

^a^Two leaves were sprayed with the liquid culture once a day three times before inoculation on the 4^th^ day. Each leaf was inoculated at five sites, and the disease incidence and lesion diameter were recorded after 3 days.

^b^Spore suspension mixed with the same amount of extract was incubated at 28°C for 4 h. Data were means of three replicates.

### Isolation of inhibitor and nature of its inhibition

Cultures of *E. brefeldianum* were each extracted with a different solvent. The extracts were tested for their ability to inhibit spore germination of *A. brassicicola* after solvent evaporation with the exception of water extract which was tested directly without evaporation treatment. Germination rates were recorded after incubation for 4 h. The result showed that spore germination was inhibited nearly completely in water extract and completely in ethanol and methanol extracts. Extracts of acetone, ethyl acetate, ether and chloroform were essentially non-inhibitory, supporting 89 to 97% germination in comparison with 97% in water control (Table 2). When germination rates were recorded after incubation for 24 h, *A. brassicicola* germinated 67, 79 and 20% in extracts of water, ethanol and methanol, respectively. Methanol was, therefore, selected for extraction of the inhibitor from the freeze dried powder of *E. brefeldianum* for subsequent studies.

**Table 2:** **Effectiveness of different solvents in extracting inhibitory substances from freeze-dried powder of liquid culture of** ***Eupenicillium brefeldianum*** **against germination of** ***Alternaria brassicicola*** **conidia**

	Germination (%)^a^		
	Extract		Control
Solvent	4 h	24 h	4 h
Water	2	67	100
Ethanol	0	79	99
Methanol	0	20	100
Acetone	89		99
Ethyl acetate	96		99
Ether	89		91
Chloroform	97		96
Water (control)	97		

^a^Spore suspension mixed with the same amount of extract was incubated at 28°C. Data were means of three replicates.

To determine if the antifungal effect of the inhibitor is fungicidal or fungistatic, conidial suspension of *A. brassicicola* was mixed with culture extract of *E. brefeldiamun*. The mixture was incubated at 28°C for 24 or 48 h before centrifugation at 1500 × g for 5 min to remove the inhibitor. The conidial suspension was then spread on water agar and incubated at 28°C for 4 h to test their viability. The result showed that after exposure of conidia to the inhibitor for 24 and 48 h, essentially all of them became non-viable (Table 3), indicating that the antifungal effect of the inhibitor was fungicidal.

**Table 3:** **Germination of conidia of** ***Alternaria brassicicola*** **on water agar after exposure to culture extract of** ***Eupenicillium brefeldianum*** **for 24 or 48 h**

Exposure time	Germination (%)^a^		
(h)	Extract	Water agar	Control
24	0	7	99
48	0	4	100

^a^Spore suspension mixed with the same amount of extract was incubated at 28°C for 4 h. Data were means of three replicates.

### Effect of pH on the inhibitory activity

The inhibitory effect of the culture extract of *E. brefeldianum* against germination of *A. brassicicola* conidia was not affected when its pH was adjusted from the original 4.0 to 3.0. At pH 3.0, the germination rate was decreased from 91% in the control to 0% (Table 4). When the pH was increased from 4.0 to 5.0, the germination rate was increased from 0 to 59%. At pH ranging from 6.0 to 10.0, the extract was no longer inhibitory.

**Table 4:** **Conidial germination of** ***Alternaria brassicicola*** **in culture extract of** ***Eupenicillium brefeldianum*** **adjusted to various pH values**

	Germination (%)^a^	
pH value after adjustment	Extract	Water (control)
3.0	0	91
4.0 (original)	0	89
5.0	59	91
6.0	98	94
7.0	98	93
8.0	93	93
9.0	95	87
10.0	91	93

^a^Spore suspension mixed with the same amount of extract was incubated at 28°C. Data were means of three replicates.

### Effect of the extreme pH and high temperature on the stability of the fungicidal substance

To determine the effect of extreme pH on the stability of the fungicidal substance produced by *E. brefeldiamun*, its culture extract was adjusted to pH 2.0 or 12.0 for 24 h before being adjusted back to the original pH of 4.0. The result showed that the fungicidal substance was very stable at pH 2.0 and 12.0. After 24 h exposure, the extract still inhibited conidial germination of *A. brassicicola* completely in comparison with the 98% germination in the water control (Table 5).

**Table 5:** **Conidial germination of** ***Alternaria brassicola*** **in culture extract of** ***Eupenicillium brefeldianum*** **adjusted to pH value of 2.0 or 12.0 for 24 h before being adjusted back to the original pH of 4.0**

pH	Germination (%)^a^
2.0	0
12.0	0
4.0 Extract without treatment	0
4.0 Water (control)	98

^a^Spore suspension mixed with the same amount of extract was incubated at 28°C for 4 h. Data were means of three replicates.

To study the effect of high temperature on the stability of the fungicidal substance, the extract was treated at 60, 80 or 100°C for 30 min or autoclaved for 15 min. The result showed that the fungicidal substance was very stable at temperature ranging from 60 to 100°C (Table 6). After 30 min exposure, the extract still caused complete inhibition of conidial germination of *A. brassicicola* in comparison with the 97% germination in the water control. Even after autoclaving for 15 min, the inhibitory effect of the fungicidal substance still remained unaffected.

**Table 6:** **Effect of high temperature treatment of culture extract of** ***Eupenicillium brefeldianum*** **on its inhibitory activity against germination of** ***Alternaria brassicicola*** **conidia**

Treatment^a^	Germination (%)^b^
60	0
80	0
100	0
Autoclave	0
Non-treated extract	0
Water (control)	97

^a^Extract was treated at 60, 80 or 100°C for 30 min or autoclaved for 15 min.

^b^Spore suspension mixed with the same amount of extract was incubated at 28°C for 4 h. Data were means of three replicates.

### Effect of the adsorptive material treatment on inhibitory effect of the fungicidal substance

When the culture extract of *E. brefeldianum* was shaken with cation exchange resins or anion exchange resins, the fungicidal substance still inhibited the germination of *A. brassicicola* completely in comparison with 98% germination in water control (Table 7). However, after shaking with activated charcoal, the fungicidal substance was no longer inhibitory to spore germination. The germination rate increased from 0 to 99%.

**Table 7:** **Effect of treatment of culture extract of** ***Eupenicillium brefeldianum*** **with cation exchange resins, anion exchange resins or activated charcoal on its inhibitory activity against germination of** ***Alternaria brassicicola*** **conidia**

Treatment^a^	Germination (%)^b^
Extract without treatment	0
Cation exchange resins	0
Anion exchange resins	0
Activated charcoal	99
Water (control)	98

^a^The pH value of the treated extract was adjusted to the original pH of 4 before spore germination test.

^b^Spore suspension mixed with the same amount of extract was incubated at 28°C for 4 h. Data were means of three replicates.

## Discussion

Since soil contains abundant and diverse microorganisms (Alexander 1977), ability to colonize plant tissues under such environment is a good indication that *E. brefeldianum* possesses a strong competitive saprophytic ability (Garrett 1970). This may explain its widespread occurrence in soil (Keller and Bidochka 1998; Souza-Motta et al. 2003). The fungus produced a stable fungicidal substance when grown in broth prepared from the same vegetable tissues used in the soil amendment. This indicates the possibility of production of this fungicidal substance by *E. brefeldianum* during its colonization of plant tissues in soil. It is conceivable that suppression of substrate competitors by this fungicidal substance may account at least in part for the strong competitive saprophytic ability of this fungus in soil. Such possibility deserves further investigation.

The fungicidal substance produced by *E. brefeldianum* was very stable under high temperature and high or low pH, indicating that the compound may last very long in nature after being applied to plant leaves. This may explain why the fungicidal compound was very effective in controlling leaf spots of mustard cabbage caused by *A. brassicicola*. Although the compound was effective in controlling leaf spots of mustard cabbage in the greenhouse, its possibility of being developed into a commercial product is far from certain. Its disease control ability in the field and its toxicity to the non-target organisms in nature remain to be investigated.

The fungicidal substance produced by *E. brefeldianum* was very effective at pH 3.0 to 4.0, partially effective at pH 5.0, but not at pH ranging from 6.0 to 10.0. Several antibiotics have been shown to be more active against microorganisms in acidic medium than in the more alkaline medium (Poala et al. 1970).

Results from this study showed that the inhibitory substance produced by *E. brefeldianum* was fungicidal. The fungicidal substance in the freeze dried powder of the culture was extractable with water, ethanol and methanol, but not acetone, ethyl acetate, ether and chloroform, indicating that it is soluble in polar solvents but not soluble in non-polar solvents. The ion exchange resin test suggested that the compound did not have charges on its molecule.

## Conclusion

Results from this study suggest that *E. brefeldianum* possesses a strong competitive saprophytic ability because of its ability to produce a fungicidal substance during its colonization of plant tissues in soil. This may explain the widespread occurrence of this fungus in nature.
